# Blood-Based MicroRNAs in Psychotic Disorders—A Systematic Review

**DOI:** 10.3390/biomedicines11092536

**Published:** 2023-09-14

**Authors:** Ștefania-Alexandra Grosu, Maria Dobre, Elena Milanesi, Mihail Eugen Hinescu

**Affiliations:** 1 Faculty of Medicine, Carol Davila University of Medicine and Pharmacy, 050474 Bucharest, Romania; stefi.grosu@gmail.com (Ș.-A.G.); mhinescu@yahoo.com (M.E.H.); 2 Victor Babes National Institute of Pathology, 050096 Bucharest, Romania; maria_dobre70@yahoo.com

**Keywords:** miRNAs, blood, psychotic disorders

## Abstract

Psychotic disorders are a heterogenous class of mental illness, with an intricate pathophysiology, involving genetics and environmental factors, and their interaction. The identification of accessible biomarkers in bodily systems such as blood may lead to more accurate diagnosis, and more effective treatments targeting dysfunctional pathways, and could assist in monitoring the disease evolution. This systematic review aims to highlight the dysregulated microRNAs (miRNAs) in the peripheral blood of patients with psychotic disorders. Using the PRISMA protocol, PubMed and Science Direct databases were investigated and 22 articles were included. Fifty-five different miRNAs were found differentially expressed in the blood of psychotic patients compared to controls. Seventeen miRNAs (miR-34a, miR-181b, miR-432, miR-30e, miR-21, miR-137, miR-134, miR-7, miR-92a, miR-1273d, miR-1303, miR-3064-5p, miR-3131, miR-3687, miR-4428, miR-4725-3p, and miR-5096) were dysregulated with the same trend (up- or down-regulation) in at least two studies. Of note, miR-34a and miR-181b were up-regulated in the blood of psychotic patients in seven and six studies, respectively. Moreover, the level of miR-181b in plasma was found to be positively correlated with the amelioration of negative symptoms. The panel of miRNAs identified in this review could be validated in future studies in large and well-characterized cohorts of psychotic patients.

## 1. Introduction

Psychotic disorders are mental illnesses characterized by psychotic symptoms, which are generally described as significantly altered or distorted perceptions of reality. Although their median lifetime prevalence has been estimated to be only 7.49 per 1000 [1], psychotic disorders cause substantial functional impairment in affected individuals. Schizophrenia, whose prevalence was 24 million in 2019, represents one of the top 15 leading causes of disability worldwide [2] with patients experiencing a significantly lower quality of life than unaffected individuals [3].

Unless psychotic symptoms appear because of drugs or substance use, or other medical conditions such as brain tumors, viral infections, or metabolic disorders [4], their etiology has not been clearly defined yet. What is certain is that a complex interplay between genetic and environmental factors occurs, playing an important role in the onset and progression of such diseases [5,6].

Data on the genetic component of psychiatric disorders is mainly derived from twin studies. A recent work based on the nationwide Danish Twin Register has estimated that the heritability of psychotic disorders is 73%, while multiple studies reported the heritability of schizophrenia and bipolar disorder with a value of approximately 80% [7,8,9] and 70%, respectively [10].

The environmental factors associated with psychotic disorders are heterogeneous. Childhood trauma and infections have been extensively researched and frequently linked to both schizophrenia and bipolar disorder, while obstetric complications, birth in the winter or spring, migration, and urban living are risk factors mainly associated with schizophrenia, while their role in bipolar disorder is not [11]. Uncertain results have been obtained on Vitamin D, with many studies reporting lower serum Vitamin D levels in patients suffering from psychotic disorders compared to healthy individuals [12], without explaining whether this is a causal relationship or an effect of poorer patients’ lifestyle choices. Moreover, other studies have shown that both neonatal Vitamin D deficiency and excess seem to increase the risk of developing schizophrenia during later life [13,14].

From the clinical point of view, psychotic disorders are characterized by dysfunctions in at least one of the following areas: hallucinations, delusions, disorganized thinking or speech, abnormal motor behavior, and negative symptoms. The duration and number of symptoms of affected individuals differentiate between the disorders categorized under the term “Schizophrenia Spectrum and Other Psychotic Disorders”, in the DSM-5 [15]. Table 1 presents the disorders included in the category of psychotic disorders, as classified by the DSM-5.

At present, psychotic disorders are diagnosed exclusively based on clinical features, and while clinicians have become very skilled at assessing symptoms, the lack of biological tests represents an important limit in psychiatry.

Over the past two decades, an extraordinary effort has been made to identify biomarkers that are significantly associated with either a specific symptom or a cluster of symptoms from the category of psychotic disorders. These could be useful for both diagnostic and disease monitoring purposes, as well as for predicting treatment response, and potential side effects to certain antipsychotics, so that even initial treatment can directly bypass individual genetic resistance mechanisms.

Moreover, these biomarkers could help to predict the conversion to psychosis in individuals from high-risk groups.

The search for biomarkers has been oriented towards two main areas: central biomarkers, where the state of the brain is being analyzed using various neuroimaging methods, and peripheral biomarkers, where specific molecules from different peripheral tissues are quantified, in an attempt to correlate them with certain symptoms.

The most investigated central biomarker associated with schizophrenia is dopaminergic hyperactivity at the D2 dopamine receptor. Along with studies using SPECT (single-photon emission computed tomography), detecting a hyperdopaminergic state both in first-episode psychosis and subsequent episodes [16], pharmacological studies on D2 antagonists have been conducted, correlating higher occupancy of the receptors with clinical improvement of psychotic symptoms [17,18].

A newer hypothesis of the pathophysiological process of psychotic disorders is the NMDA receptor hypofunction, which causes a hyperglutamatergic state in specific brain regions such as the striatum [19], or the hippocampus [20]. Subsequently, glutamatergic antagonism increases dopamine release in the striatum and cerebral cortex. Therefore, an interaction between the dopaminergic and glutamatergic systems could best account for the multiple dimensions of psychotic symptoms [20].

Another theory involving the hippocampus is hippocampal hyperactivity, reflected in an increased blood flow, using various magnetic resonance sequences [21,22]. Other potential neuroimaging biomarkers can detect neuroinflammation using PET [23], and grey matter reduction [24], which could be associated. Neuroimaging biomarkers have the advantage that they can directly analyze the state of the brain in different phases of the disorder; however, they are expensive and require the collaboration of patients who, in many cases, are vulnerable or not cooperative. Therefore, it would be difficult to conclude a potential neural correlation, due to the heterogeneity of the results.

Peripheral biomarkers can be investigated in more accessible biological samples such as blood, plasma, saliva, or cerebrospinal fluid and can be represented by DNA methylation [25], histone modifications [26], mRNA, or the identification of various types of noncoding RNA, such as microRNA (miRNA), or long noncoding RNA [27]. Cytokines involved in inflammation can also be identified in peripheral tissues of psychotic patients, as well as abnormally functioning blood cells [28,29]. Unlike central biomarkers, peripheral biomarkers have the great advantage that they are easy to obtain, and do not require the patients to cooperate for long periods of time. However, they are greatly dependent on any medication that the patient is administered, and can also be influenced by recent bacterial or viral infections.

MicroRNAs are small noncoding RNA, with an average length of 22 nucleotides. The main role of miRNAs is regulating gene expression post-transcriptionally, by binding to mRNA in the cytoplasm of cells, and consequently either blocking protein expression or causing the degradation of mRNA [30,31]. A single molecule of miRNA can target a large array of up to hundreds of different unique mRNAs [32].

miRNA can be found both intracellularly, and in the extracellular compartment, which facilitates their identification in tissues such as cerebrospinal fluid, peripheral whole blood, plasma, or serum [33]. Of note, extracellular miRNAs have been found to be more resistant to endogenous RNase [34] than intracellular miRNAs, and some studies show that extracellular miRNAs remain stable even in the case of pH alterations [35].

This review aims to provide a comprehensive assessment of the current progress regarding the potential value of blood-based miRNAs in psychotic disorders by centralizing the published results on the topic and by verifying whether any specific miRNA correlates with certain clinical psychotic features. Although other studies have reviewed the various epigenetic mechanisms involved in psychotic disorders [36,37,38,39,40,41], not all the studies have used a systematic approach. This work represents the most recent systematic review that summarizes the roles of blood-based miRNAs found consistently abnormally expressed in psychotic disorders, including in the first episode of psychosis.

## 2. Methods

This systematic review was carried out according to the Preferred Reporting Items for Reviews and Meta-Analyses (PRISMA) 2020 guideline [42].

### 2.1. Data Sources, Search Strategy, and Eligibility Criteria for Article Inclusion

**Figure 1:**
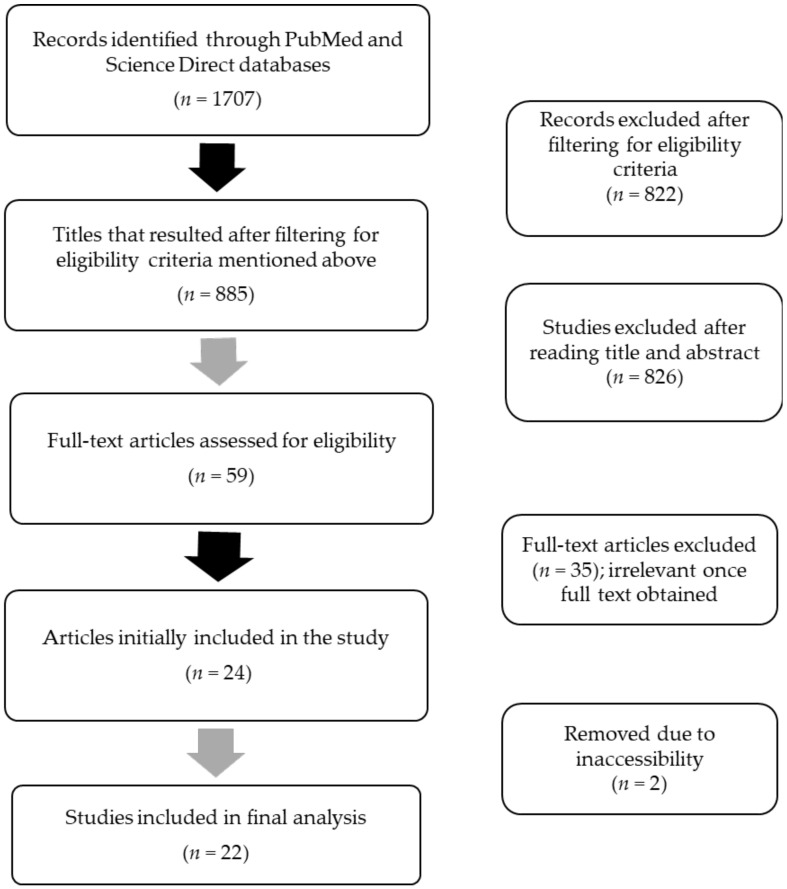
PRISMA flow diagram of the studies included in this review.

The database search was conducted between December 2022 and January 2023, and the articles included in this review were selected from the PubMed and Science Direct databases. The keywords used in each database were the following: (psychosis OR schizophrenia) AND (miRNA OR microRNA). This review has been registered with PROSPERO (registry number CRD42023429319). The inclusion criteria for the studies reported in this review were as follows: (i) studies investigating miRNA expression exclusively in human subjects and including both sexes; (ii) the article type being only original research articles; (iii) the study design being case-control type; (iv) the study having been originally written in English, or any other language, with an available English translation; (v) articles published between January 2010 and January 2023. This review is based on 22 remaining articles that fulfilled the eligibility criteria, as shown in Figure 1.

### 2.2. Data Extraction and Risk of Bias Assessment

The data extracted from each publication were: authors, year of publication, sample size, diagnoses, type of sample, presence/absence of psychotropic medication, miRNAs dysregulation type (significant up- or down-regulation). The possibility of bias in the design and analysis of each study included was assessed by two different evaluators using the QUADAS-2 (Quality Assessment of Diagnostic Accuracy Studies) tool.

## 3. Results

The PRISMA flow diagram for the selection of articles included in this study is presented in Figure 1.

A total number of 22 studies were included in this review. All of them included patients with a diagnosis of either first-episode psychosis (311 subjects from 7 studies), schizophrenia (1762 subjects from 20 studies), or bipolar disorder (31 subjects, 1 study). A total of 842 patients from 16 different studies were either treatment-naive or had not been administered any psychotropic medication for at least 3 months before being included in the studies. The other 1262 individuals had been exposed to antipsychotic medication before being included.

The analyzed miRNAs were isolated from the following tissues: peripheral blood mononuclear cells (PBMCs; 7 studies), plasma (5 studies), whole blood (5 studies), serum (3 studies), serum extracellular vesicles (1 study), and both plasma and PBMCs (1 study).

Quantification of miRNAs was performed using qRT-PCR (12 studies), microarray followed by qRT-PCR validation (6 studies), or RNA sequencing followed by qRT-PCR validation (4 studies). Details of the 22 study characteristics are summarized in Table 2. The diagram showing the bias detected in the different domains and the overall bias of studies included is presented in Supplementary Materials (Table S1).

After centralizing the results, the expression of 20 miRNAs has been identified to be significantly dysregulated in psychotic patients compared to controls, in at least two different studies. Among them, 17 miRNAs were found to be dysregulated in more than one study with the same trend (up- or down-regulation) (Table 3).

Three miRNAs (miR-195, miR-346, and miR-132) have shown contrasting results in different studies.

Only 3 of the studies included in this review analyzed the presence of correlations between miRNA levels and specific psychotic symptoms (Table 4). Lai et al. (2011) concluded that miR-449 levels were positively correlated with negative symptoms on the PANSS questionnaire [44]. Song et al. (2014) identified another miRNA correlating with negative symptoms—in this case, miR-181b was positively correlated with the improvement of negative symptoms [46]. Finally, Chen et al. (2016), stated that miR-21 was negatively correlated with improvement of positive, general psychopathology, and aggressiveness symptoms [54].

## 4. Discussion

This systematic review aimed to synthesize literature data from multiple databases, highlighting the differentially expressed miRNAs in the blood of psychotic patients compared to controls. The analysis of the included studies yielded various results identifying 65 different miRNAs. Among them, 17 were found in multiple studies, with a consistently abnormal expression in the same direction. Notably, miR-34a and miR-181b were found up-regulated in the blood of psychotic patients compared to controls in seven and six independent studies, respectively, suggesting that these two miRNAs could represent possible diagnostic biomarkers. The increased expression of these miRNAs was observed in different blood components, including PBMCs, plasma, and serum of psychotic patients. In addition, miR-181b was also found dysregulated in whole blood [56,62].

miR-34 is a family of miRNAs encoded by chromosome 1p36, consisting of 3 members, miR-34a, b, and c, and with the highest expression in the brain. miR-34a has been the most studied molecule of the miR-34 family. In the central nervous system, miR-34 regulates neural stem cell differentiation, and, when overexpressed, contributes to neurite elongation. Its expression is dependent on p53 proteins, creating a feedback loop that is more active during neural development [65,66].

In psychiatric disorders, multiple studies identified miR-34a to be differentially expressed in postmortem cerebral tissues of patients with schizophrenia or bipolar disorder, compared to a healthy control group [67,68]. Additionally, studies investigating miR-34′s expression in peripheral blood mononuclear cells identified higher levels in patients with schizophrenia, major depressive disorder, or Alzheimer’s disease, compared to control groups. Consistent with these results, one study concluded that a 12-week treatment with escitalopram significantly decreased the miR-34c expression in peripheral blood [69].

miR-181 is a family of microRNAs with expression predominantly in B cells, the retina, and the brain [70]. The mature products of the miR-181 precursor are miR-181a, b, c, and d. The articles included in this review have highlighted the up-regulation of miR-181b in psychotic disorders in six different studies.

In 2008, Beveridge et al. found that there was significant up-regulation of miR-181b in the superior temporal gyrus of patients with schizophrenia, compared to controls [71]. The same study identified that the calcium sensor gene visinin-like 1 (*VSNL1*) and the ionotropic AMPA glutamate receptor subunit (*GRIA2*) genes were down-regulated as an effect of the aberrant expression of the miRNA. These results are consistent with the conclusions of this review, rendering miR-181b a miRNA playing an important role in the development of psychiatric disorders, specifically psychosis. Indeed, our review identified a study showing that the level of miR-181b in plasma was found to positively correlate with the amelioration of negative symptoms, suggesting that, if validated in a larger cohort, this miRNA could be a biomarker for monitoring the disease evolution and the response to therapy.

Although miR-432 has been studied less than miR-34a and miR-181b some interesting results showing a putative role in psychiatric disorders have been found. Zhang et al. identified miR-432 to be crucial in mediating the antidepressant effect of the molecule *ADAR-1* (Adenosine deaminase acting on RNA1), specifically through brain-derived neurotrophic factor (*BDNF*) [72]. Moreover, a recent study [73], found that miR-432 was down-regulated in peripheral extracellular vesicles of adolescents with major depressive disorder or anxiety compared to controls.

The other two miRNAs found significantly up-regulated in the blood of psychotic patients in three different studies are miR-30e and miR-21. miR-30 is a family consisting of 5 members, miR-30a, b, c, d and e. A postmortem study that included patients with Alzheimer’s disease (AD), stated that miR-30e was up-regulated in the hippocampus at Braak stages III/IV of non-demented and early AD subjects and that overexpression of the molecule increased the levels of superoxide dismutase, glutathione, and glutathione-peroxidase, and decreases ROS levels by inhibiting TGF-β [74]. Although our review has identified an up-regulation of this miRNA in the plasma of psychotic patients, Perkins et al. [75], identified miR-30e to be down-regulated in the prefrontal cortex of patients with schizophrenia and schizoaffective disorder. This opposite trend is not surprising, since the relationship between blood and brain miRNA expression activity is not well understood and it is known that the miRNA blood–brain correlations are region-specific [76].

miR-21, identified as up-regulated in PBMCs of psychotic patients in three studies, is a miRNA that generally targets tumor suppressor genes, and has been associated with multiple types of cancers [77]. In the central nervous system, miR-21 has been correlated particularly with disorders involving inflammation, such as Alzheimer’s disease or multiple sclerosis [78]. An interesting aspect regarding miR-21 is that it has been found to be both ubiquitous in many different cell types and overexpressed in various disease states. Therefore, its lack of specificity poses a problem when studies try to correlate it with certain disorders [79].

Three articles included in this review found abnormal levels of miR-195 in the peripheral blood of psychotic patients; however, two of them found it to be overexpressed, while the third one concluded it was down-regulated in patients compared to controls. This discrepancy could be explained by the fact that the latter study included patients previously exposed to antipsychotic medication, and the down-regulation of miR-195 could be an effect of the treatment. Multiple studies analyzing microRNA molecules from postmortem brain samples, found miR-195 to be consistently up-regulated [80,81]. miR-195 has been found to have a role in regulating *BDNF* [82], suggesting that, as well as the role of diagnostic biomarker, it is also involved in the mechanisms underlying psychotic disorders.

Another miRNA with strong evidence of abnormal values in postmortem brain tissue is miR-7: multiple studies have reported its up-regulation in the dorsolateral prefrontal cortex of patients with schizophrenia compared to unaffected patients [67,75,80]. An article by Zhao et al. (2020), investigated the physiological and pathological roles of miR-7 in the central nervous system. In the field of psychiatric disorders, miR-7 has been found to interfere with the *SHANK3* domains, involved in memory and synaptic plasticity. miR-7 also has different roles in the pathogenesis of neurodegenerative diseases, such as Alzheimer’s disease or Parkinson’s disease, and neuroinflammation [83].

Consistent with a broader review of miRNAs in psychiatric disorders, the most frequently abnormally expressed miRNAs in psychotic disorders, such as miR-34a, miR-181b, or miR-30e, were specific to this diagnostic class, and could not be identified in other psychiatric disorders, such as affective disorders or addictions [84]

After the discovery of miRNAs in 1993 [85], numerous studies examining their role in various disorders have been conducted. These short noncoding RNA have been particularly studied in the field of oncology, as new and precise biomarkers are needed for the improvement of therapeutic and monitoring protocols. Although psychiatry, unlike oncology, does not yet rely on any objectively measurable parameters for diagnosis, monitoring, or predicting treatment response, identifying miRNAs that could potentially play such a role, would be crucial for facilitating the development of this field.

The results presented in this review have some limitations: first, some of the studies included patients treated with antipsychotics, and other studies only treatment-naive patients. The presence of a specific therapeutic regimen could explain the identification of some miRNAs with opposite results in their expression in different studies. Second, this review summarizes the evidence identified in different blood components (whole blood, PBMC, plasma, and serum) obtained with different methodologies, and although this aspect has been highlighted in the manuscript, the results must be carefully interpreted. Moreover, most of the included studies were conducted on the Asian population, whose genetic background can be different from those of other ethnicities.

Although many studies have investigated the expression of specific miRNAs in psychiatric disorders, few of them have accounted for their varying expression throughout an individual’s lifetime. Future studies need to be conducted, focusing on miRNA expression in different age groups, both in healthy individuals and in patients suffering from psychiatric disorders.

Moreover, the fact that the same miRNAs were found to be abnormally expressed in multiple psychiatric disorders may indicate that the current classification of these disorders is not consistent with their underlying neurobiological mechanisms. Therefore, future research should be directed toward identifying epigenetic correlates to transdiagnostic dimensions of psychiatric disorders, aiming to verify whether current psychiatric nosology is accountable for the failure to identify consistent biomarkers corresponding to these classes.

Another direction for future studies in the field of miRNAs and, more generally, epigenetic studies in psychiatric disorders, is conducting longitudinal studies with large community cohorts, as opposed to case-control, cross-sectional studies. These designs could reveal a more accurate image of miRNA expression both inter- and intraindividually, as well as the molecules and correspondent genes involved in the various stages of psychiatric disorders.

Although the field of peripheral blood biomarkers in psychiatric disorders has been developing for the last decade, future studies are needed to clarify the pros and cons of blood as tissue for investigating miRNAs, and better explain the brain-blood correlations. Since the relationship between brain and blood miRNA expression is not well understood the research should focus on the identification of how and if the brain-blood miRNA correlations are useful not only as biomarkers but also reflect the etiological mechanisms of psychotic disorders.

Once these issues are clarified, miRNAs could be used as biomarkers for various aspects of psychiatric disorders, such as the risk of developing certain disorders, their prognosis, identifying patients’ susceptibility to different compounds, as well as symptom remission.

## Figures and Tables

**Table 1:** Schizophrenia Spectrum and Other Psychotic Disorders, as classified by the DSM-5 (American Psychiatric Association, 2013 [15]).

Schizophrenia Spectrum and Other Psychotic Disorders
Schizotypal personality disorder
Delusional disorder
Brief psychotic disorder
Schizophreniform disorder
Schizophrenia
Schizoaffective disorder
Psychotic disorders induced by another condition: − Substance or medication-induced − Due to another medical condition
Catatonia
Other specified schizophrenia spectrum and other psychotic disorder
Unspecified schizophrenia spectrum and other psychotic disorder

**Table 2:** Summary table of the characteristics and results of the studies included in this review.

No.	Author (Year)	Diagnosis	Tissue	Method	Intervention	miRNAs Identified	Number of Patients
1	Gardiner et al. (2011) [43]	Schizophrenia	PBMCs	Microarray followed by qRT-PCR	All patients previously received antipsychotic treatment	mir-31 ↓ mir-431 ↓ mir-433 ↓ mir-107 ↓ mir-134 ↓ mir-99b ↓ mir-487b ↓	112 SCZ; 76 controls
2	Lai et al. (2011) [44]	Schizophrenia	PBMCs	qRT-PCR	All patients previously received antipsychotic treatment	miR-34a ↑ miR-449a ↑ miR-564 ↑ miR-548d ↑ miR-572 ↑ miR-652 ↑ miR-432 ↓	90 SCZ; 60 controls
3	Shi et al. (2012) [45]	Schizophrenia	Serum	qRT-PCR	All patients previously received antipsychotic treatment	miR-181b ↑ miR-219-2-3p ↑ miR-346 ↑ miR-1308 ↑ miR-92a ↑ miR-195 ↓ miR-17 ↓	115 SCZ; 40 controls
4	Song et al. (2014) [46]	Schizophrenia	Plasma	qRT-PCR	None, or at least 3 months with no psychotropic medication.	miRNA-181b ↑ miRNA-30e ↑ miRNA-34a ↑ miRNA-7 ↑	20 SCZ; 20 controls
5	Fan et al. (2015) [47]	Schizophrenia	PBMCs	Microarray followed by qRT-PCR	None, or at least 3 months with no psychotropic medication.	miR-1273d ↑ miR-1303 ↑ miR-21 ↑ miR-3064-5p ↑ miR-3131 ↑ miR-3687 ↑ miR-4428 ↑ miR-4725-3p ↑ miR-5096 ↑	55 SCZ; 28 controls
6	Yu et al. (2015) [48]	Schizophrenia	PBMCs	Microarray followed by qRT-PCR	105 treatment-naive patients	miR-132 ↓ miR-134 ↓ miR-1271 ↓ miR-664 ↓ miR-200c ↓ miR-432 ↓	105 SCZ; 130 control
7	Sun et al. (2015a) [49]	Schizophrenia	Plasma and PBMCs	qRT-PCR	No antipsychotic treatment, or at least 3 months with no psychotropic medication.	miR-132 ↑ plasma miR-195 ↑ plasma miR-30e ↑ plasma miR-7 ↑ plasma miR-212 ↑ PBMC miR-34a ↑ PBMC miR-30e ↑ PBMC	25 SCZ; 13 control
8	Sun et al. (2015b) [50]	Schizophrenia	Plasma	qRT-PCR	No antipsychotic treatment, or at least 3 months with no psychotropic medication.	miR-30e ↑ miR-181b ↑ miR-34a ↑ miR-346 ↑ miR-7 ↑	61 SCZ; 62 control
9	Wei et al. (2015) [51]	Schizophrenia	Plasma	RNA Sequencing followed by qRT-PCR	164 drug-naive patients; 400 patients with previous antipsychotic treatment	miR-130b ↑ miR-193a-3p ↑	564 SCZ; 400 control
10	Lai et al. (2016) [52]	Schizophrenia (acute state)	PBMCs	qRT-PCR	4 drug-naive patients; 44 patients with previous antipsychotic treatment	miR-34a ↑ miR-564 ↑ miR-548d ↑ miR-449a ↑	48 SCZ; 37 control
11	Camkurt et al. (2016) [53]	Schizophrenia (Active psychotic episode)	Whole blood	qRT-PCR	3 drug-naive patients; 13 patients with previous antipsychotic treatment	miR-9-5p ↑ miR-29a-3p ↑ miR-106b-5p ↑ miR-125a-3p ↑ miR-125b-3p ↑	16 SCZ; 16 control
12	Chen et al. (2016) [54]	Schizophrenia	PBMCs	Microarray followed by qRT-PCR	None, or at least 3 months with no psychotropic medication.	miR-1273d ↑ miR-1303 ↑ miR-21 ↑ miR-3064-5p ↑ miR-3131 ↑ miR-3687 ↑ miR-4428 ↑ miR-4725-3p ↑ miR-5096 ↑	82 SCZ; 43 controls
13	Liu et al. (2017) [55]	Schizophrenia	PBMCs	qRT-PCR	None, or at least 3 months with no psychotropic medication.	miR-181b-5p ↑ miR-21-5p ↑ miR-195-5p ↑ miR-137 ↑ miR-34a-5p ↑ miR-346 ↓	20 first-episode schizophrenia 19 schizophrenia 50 controls
14	Ma et al. (2018) [56]	First-onset schizophrenia	Whole blood	RNA sequencing followed by qRT-PCR	All patients were drug-naive.	miR-22-3p ↑ miR148b-5p ↑ miR-181a-5p ↑ miR-181b-5p ↑ miR-199b-5p ↑ miR-92a-3p ↑	10 first-onset SCZ; 10 control (RNA sequencing) and 44 SCZ; 44 controls (qRT-PCR)
15	He et al. (2019) [57]	Schizophrenia	Serum	qRT-PCR	All patients had previously received antipsychotic treatment	miR-34a-5p ↑ miR-449a ↑ miR-432-5p ↓	40 SCZ; 40 control
16	Wang et al. (2019) [58]	Schizophrenia	Serum	Microarray followed by qRT-PCR	59 treatment-naive patients 3 clinically cured patients	miR-320a-3p ↓ miR-320b ↓	3 treatment-naive SCZ, 3 clinically cured SCZ and 3 control (Microarray analysis); 59 SCZ and 60 control (qRT-PCR validation)
17	Zhao et al. (2019) [59]	First-episode psychosis and Schizophrenia	Plasma	Microarray followed by qRT-PCR	17 FEP patients with no history of antipsychotic treatment for longer than 16 weeks 21 SCZ patients with previous antipsychotic treatment	miR-223-3p ↑ FEP, SCZ miR-6131 ↑FEP	17 FEP 17 control and 21 SCZ; 21 control
18	Du et al. (2019) [60]	Schizophrenia	Serum-derived exosomes	RNA sequencing followed by qRT-PCR	106 drug-free patients 43 patients with previous antipsychotic treatment	miR-206 ↑ miR-619 ↑ miR-144-3p ↓	49 drug-free first-episode SCZ; 46 controls (RNA sequencing) 100 SCZ (57 first-episode, drug-free patients and 43 chronically treated patients); 100 controls (qRT-PCR)
19	Horai et al. (2020) [61]	Schizophrenia	Whole blood	qRT-PCR	All patients were under chronic antipsychotic treatment	miR-19b ↑	22 SCZ; 19 control
20	Gou et al. (2021) [62]	First-episode schizophrenia	Whole blood	qRT-PCR	10 drug-naive patients; 113 patients had previously received antipsychotic medication	miR-181b-5p ↑	123 first-episode SCZ; 50 controls
21	Chen et al. (2021) [63]	Schizophrenia Bipolar disorder	Plasma	qRT-PCR	Medically stabilized	miR-137 ↑ SCZ, relatives miR-34b ↑ SCZ, relatives miR-34c ↑ SCZ, relatives	215 SCZ; 72 unaffected first-degree relatives of SCZ patients 31 BD; 100 controls
22	Jin et al. (2022) [64]	First-episode Schizophrenia	Whole blood	RNA sequencing followed by qRT-PCR	None	miR-9-5p ↓ miR-4467 ↑	35 FES; 60 control

**Table 3:** miRNAs found to be dysregulated in at least two studies.

**No.**	**miRNAs**	**Study (Author, Year)/Tissue**	**Expression**
1.	miR-34a	Lai et al. (2011)/PBMC [44] Song et al. (2014)/Plasma [46] Sun et al. (2015a)/PBMC [49] Sun et al. (2015b)/Plasma [50] Lai et al. (2016)/PBMC [52] Liu et al. (2017)/PBMC [55] He et al. (2019)/Serum [57]	↑ 7 studies (4 PBMC; 2 Plasma; 1 Serum)
2.	miR-181b	Shi et al. (2012)/Serum [45] Song et al. (2014)/Plasma [46] Sun et al. (2015b)/Plasma [50] Liu et al. (2017)/PBMC [55] Ma et al. (2018)/Whole blood [56] Gou et al. (2021)/Whole blood [62]	↑ 6 studies (1 PBMC; 2 Plasma; 1 Serum; 2 Whole Blood)
3.	miR-432	Lai et al. (2011)/PBMC [44] Yu et al. (2015)/PBMC [48] He et al. (2019)/Serum [57]	↓ 3 studies (2 PBMC; 1 Serum)
4.	miR-30e	Song et al. (2014)/Plasma [46] Sun et al. (2015a)/Plasma and PBMC [49] Sun et al. (2015b)/Plasma [50]	↑ 3 studies (1 PBMC; 3 Plasma)
5.	miR-21	Fan et al. (2015)/PBMC [47] Chen et al. (2016)/PBMC [54] Liu et al. (2017)/PBMC [55]	↑ 3 studies (3 PBMC)
6.	miR-137	Liu et al. (2017)/PBMC [55] Chen et al. (2021)/Plasma [63]	↑ 2 studies (1 PBMC; 1 Plasma)
7.	miR-134	Gardiner et al. (2011)/PBMC [43] Yu et al. (2015)/PBMC [48]	↓ 2 studies (2 PBMC)
8.	miR-7	Sun et al. (2015a)/Plasma [49] Sun et al. (2015b)/Plasma [50]	↑ 2 studies (2 Plasma)
9.	miR-92a	Shi et al. (2012)/Serum [45] Ma et al. (2018)/Whole Blood [56]	↑ 2 studies (1 Serum; 1 Whole Blood)
10.	miR-1273d	Fan et al. (2015)/PBMC [47] Chen et al. (2016)/PBMC [54]	↑ 2 studies (2 PBMC)
11.	miR-1303	Fan et al. (2015)/PBMC [47] Chen et al. (2016)/PBMC [54]	↑ 2 studies (2 PBMC)
12.	miR-3064-5p	Fan et al. (2015)/PBMC [47] Chen et al. (2016)/PBMC [54]	↑ 2 studies (2 PBMC)
13.	miR-3131	Fan et al. (2015)/PBMC [47] Chen et al. (2016)/PBMC [54]	↑ 2 studies (2 PBMC)
14.	miR-3687	Fan et al. (2015)/PBMC [47] Chen et al. (2016)/PBMC [54]	↑ 2 studies (2 PBMC)
15.	miR-4428	Fan et al. (2015)/PBMC [47] Chen et al. (2016)/PBMC [54]	↑ 2 studies (2 PBMC)
16.	miR-4725-3p	Fan et al. (2015)/PBMC [47] Chen et al. (2016)/PBMC [54]	↑ 2 studies (2 PBMC)
17.	miR-5096	Fan et al. (2015)/PBMC [47] Chen et al. (2016)/PBMC [54]	↑ 2 studies (2 PBMC)
18.	miR-195	Shi et al. (2012)/Serum [45] Sun et al. (2015a)/Plasma [49] Liu et al. (2017)/PBMC [55]	↑ 2 studies (1 PBMC: 1 Plasma) ↓ 1 study (1 Serum)
19.	miR-346	Shi et al. (2012)/Serum [45] Sun et al. (2015b)/Plasma [50] Liu et al. (2017)/PBMC [55]	↑ 2 studies (1 Plasma; 1 Serum) ↓ 1 study (1 PBMC)
20.	miR-132	Sun et al. (2015a)/Plasma [49] Yu et al. (2015)/PBMC [48]	↑ 1 study (1 Plasma) ↓ 1 study (1 PBMC)

**Table 4:** MicroRNAs correlating with various symptoms from the PANSS Scale.

No.	Study (Author, Year)	miRNA/Tissue	Correlation on the PANSS Scale
1.	Lai et al. (2011) [44]	miR-449/PBMCs	Positively correlated with negative symptoms
2.	Song et al. (2014) [46]	miR-181b/Plasma	Positively correlated with the amelioration of negative symptoms
3.	Chen et al. (2016) [54]	miR-21/PBMCs	Negatively correlated with the amelioration of positive symptoms, general psychopathology, and aggressiveness symptoms

## Data Availability

Not applicable.

## Supplementary Materials

The following supporting information can be downloaded at: https://www.mdpi.com/article/10.3390/biomedicines11092536/s1, Table S1. The bias detected in the different domains and the overall bias of studies.
