# Supplementary material for: Blood-Based MicroRNAs in Psychotic Disorders—A Systematic Review
Source: Biomedicines. 2023 Sep 14;11(9):2536. doi: 10.3390/biomedicines11092536 (PMC10525934; doi:10.3390/biomedicines11092536)
Supplement: Supplementary file 1 [file biomedicines-11-02536-s001.zip › biomedicines-2499832-supplementary.pdf]

Table S1. The bias detected in the different domains and the overall bias of studies.

Study	RISK OF BIAS				APPLICABILITY CONCERNS		
	Patient Selection	Index Test	Reference Standard	Flow and Timing	Patient Selection	Index Test	Reference Standard
Gardiner et al (2011)	☹	☺	☺	?	☺	☺	☺
Lai et al (2011)	☹	☺	☺	☺	☺	☺	☺
Shi et al (2012)	☹	☺	☹	?	☺	☺	☺
Song et al (2014)	☹	☺	☺	☺	☺	☺	☺
Fan et al (2015)	☹	☺	☺	☺	☺	☺	☺
Yu et al (2015)	☹	☺	☺	☺	☺	☺	☺
Sun et al (2015)	☹	☺	☺	☺	☺	☺	☺
Sun et al (2015)	☹	☺	☺	☺	☺	☺	☺
Wei et al (2015)	☹	☺	☺	☺	☺	☺	☺
Lai et al (2016)	☹	☺	☺	☺	☺	☺	☺
Camkurt et al (2016)	☹	☺	☺	☺	☺	☺	☺
Chen et al (2016)	☹	☺	☺	☺	☺	☺	☺
Ma et al (2018)	☹	☺	☺	☺	☺	☺	☺
He et al (2019)	☹	☺	☺	☺	☺	☺	☺
Wang et al (2019)	☹	☺	☺	☺	☺	☺	☺
Zhao et al (2019)	☹	☺	☺	☺	☺	☺	☺
Du et al (2019)	☹	☺	☺	☺	☺	☺	☺
Horai et al (2020)	☹	☺	☺	☺	☺	☺	☺
Gou et al (2021)	☹	☺	☺	☺	☺	☺	☺
Chen et al (2021)	☹	☺	☺	☺	☺	☺	☺
Jin et al (2022)	☹	☺	☺	☺	☺	☺	☺

☺ Low risk; ☹ High risk; ? Unclear risk.
